# Multi-stability with ambiguous visual stimuli in *Drosophila* orientation behavior

**DOI:** 10.1371/journal.pbio.2003113

**Published:** 2018-02-13

**Authors:** Franziska Toepfer, Reinhard Wolf, Martin Heisenberg

**Affiliations:** Rudolf Virchow Center, University of Wuerzburg, Germany; McGill University, Canada

## Abstract

It is widely accepted for humans and higher animals that vision is an active process in which the organism interprets the stimulus. To find out whether this also holds for lower animals, we designed an ambiguous motion stimulus, which serves as something like a multi-stable perception paradigm in *Drosophila* behavior. Confronted with a uniform panoramic texture in a closed-loop situation in stationary flight, the flies adjust their yaw torque to stabilize their virtual self-rotation. To make the visual input ambiguous, we added a second texture. Both textures got a rotatory bias to move into opposite directions at a constant relative angular velocity. The results indicate that the fly now had three possible frames of reference for self-rotation: either of the two motion components as well as the integrated motion vector of the two. In this ambiguous stimulus situation, the flies generated a continuous sequence of behaviors, each one adjusted to one or another of the three references.

## Author summary

Vision is considered an active process in humans and higher animals in which the stimulus is interpreted by the subject and can be perceived in different ways if it is ambiguous. We aimed to find out whether this also holds for lower animals, such as the fruit fly *Drosophila melanogaster*. To provide ambiguity, we exposed flies to transparent motion stimuli in a flight simulator and found their behavior to be multi-stable. These results show that the visual system of the fly can separate the individual components of a transparent motion stimulus, and that this kind of stimulus is ambiguous to the fly. The extent to which the fly shows component selectivity in its behavior depends on several properties of the stimulus, like pattern contrast and element density. The alternations between the different behaviors exhibit a stochasticity reminiscent of the temporal dynamics in human multi-stable perception.

## Introduction

Sensory stimuli serve an animal to organize its behavior. They may have a meaning or relevance. A stimulus may elicit a certain behavior, but often stimuli are ambiguous. A dark patch in the visual surround of an animal may be a potential hiding place or a predator. If the first evaluation triggers approach, this decision may have to be revised in the next moment as more information comes in. Sometimes, sensory ambiguities can be stable in time while their perception changes. This phenomenon is called multi-stable perception. It has been studied extensively over the last 50 years [1–4] and been observed not only in humans but also in nonhuman primates [5] and pigeons [6]. The capability to constantly reevaluate the current interpretation of a sensory input, with varying outcomes if it is ambiguous, may be a crucial procedure to gain more information from the stimulus so that it has evolved not only in vertebrates but also in insects.

As motion vision has been extensively investigated in *Drosophila*, visual motion stimuli are used also in the present study. The flies are exposed to a stimulus consisting of two transparent wide-field motion components moving into opposite directions. The flies can respond adaptively in different ways. Indeed, like human subjects in multi-stable perception, they alternate stochastically with this experimental design between different, but equally adaptive, behaviors. We call this the transparent panorama motion paradigm (TPMP).

Coherent angular motion of the whole panorama signals self-rotation. In the TPMP, each of the two motion components signals self-rotation. Transparent motion of two components, each one covering the whole panorama, may never occur for a freely moving subject with panoramic vision like *Drosophila*. For smaller regions of the visual field, however, transparent motion regularly occurs, for instance, when a moving subject encounters a moving object. It has been shown that *Drosophila* can restrict its behavioral responses to parts of the visual field (spatially selective visual attention) and that it also possesses mechanisms to filter out unspecific motion stimuli impinging on wide-field motion detection [7]. A study on blowflies using a two-dimensional plaid stimulus [8] also suggests that blowflies might show component selectivity with transparent motion stimuli. The two-dimensional transparent “plaid motion” stimulus [9–11] is also an example for multi-stable perception in humans. There, a moving plaid pattern can be perceived either as the coherent, nontransparent motion of the plaid, or as the transparent movements of two individual gratings moving at an angle to each other.

Little is known, however, how the fly visual system deals with completely overlapping transparent wide-field stimuli moving in the same dimension and what the behavioral response to such a stimulus might be. Traditional models of fly motion vision predict the optomotor turning response in horizontal direction to be a unimodal function of the orientation of the motion stimulus. This would include incoherently moving transparent wide-field motion stimuli [12,13], as the models assume the integration of all motion signals detected by the appropriately oriented elementary motion detectors [14]. In the case of the TPMP, the signals from the two wide-field patterns would cancel each other. A study of human transparent motion vision shows that the perception of the transparency is only present under some stimulus conditions, namely when the local motion stimuli are unbalanced [15].

Here, we show component selectivity for these patterns in *Drosophila*. How extensively the fly activates the behaviors associated with the respective components depends strongly on certain properties of the visual stimuli, particularly pattern contrast and element density. These findings may relate transparent motion processing in the *Drosophila* visual system to interactions between the figure and wide-field motion vision systems [16]. The stochasticity of the alternations between the different behaviors indicates that these alternations are generated endogenously.

## Results

### Multi-stable behavior with ambiguous visual motion stimuli

The fly's behavior was studied in the so-called flight simulator [17]. The fly was suspended at a torque meter [18] and positioned in the middle of a cylindrical light-guide arena. Its head was glued to the thorax and fixed in space. The horizontal component of the fly's angular momentum (yaw torque) was measured and transformed to generate the rotatory motion of the panorama the fly would have seen if it were free to rotate. In other words, the fly and the arena were in a negative feedback loop (closed loop) simulating the fly's horizontal angular motion.

A fly confronted in this setup with a random dots pattern uniformly covering the whole inner wall of the arena will adjust its yaw torque to a level that keeps the panorama from rotating. This behavior in the flight simulator is called optomotor balance [19]. It is assumed to be a sensory-motor equivalent of flying straight in free flight. In the flight simulator, it can be challenged by adding a rotatory bias to the panorama. Now the fly must adjust its yaw torque to a new mean value to keep the panorama from rotating.

**Fig 1:**
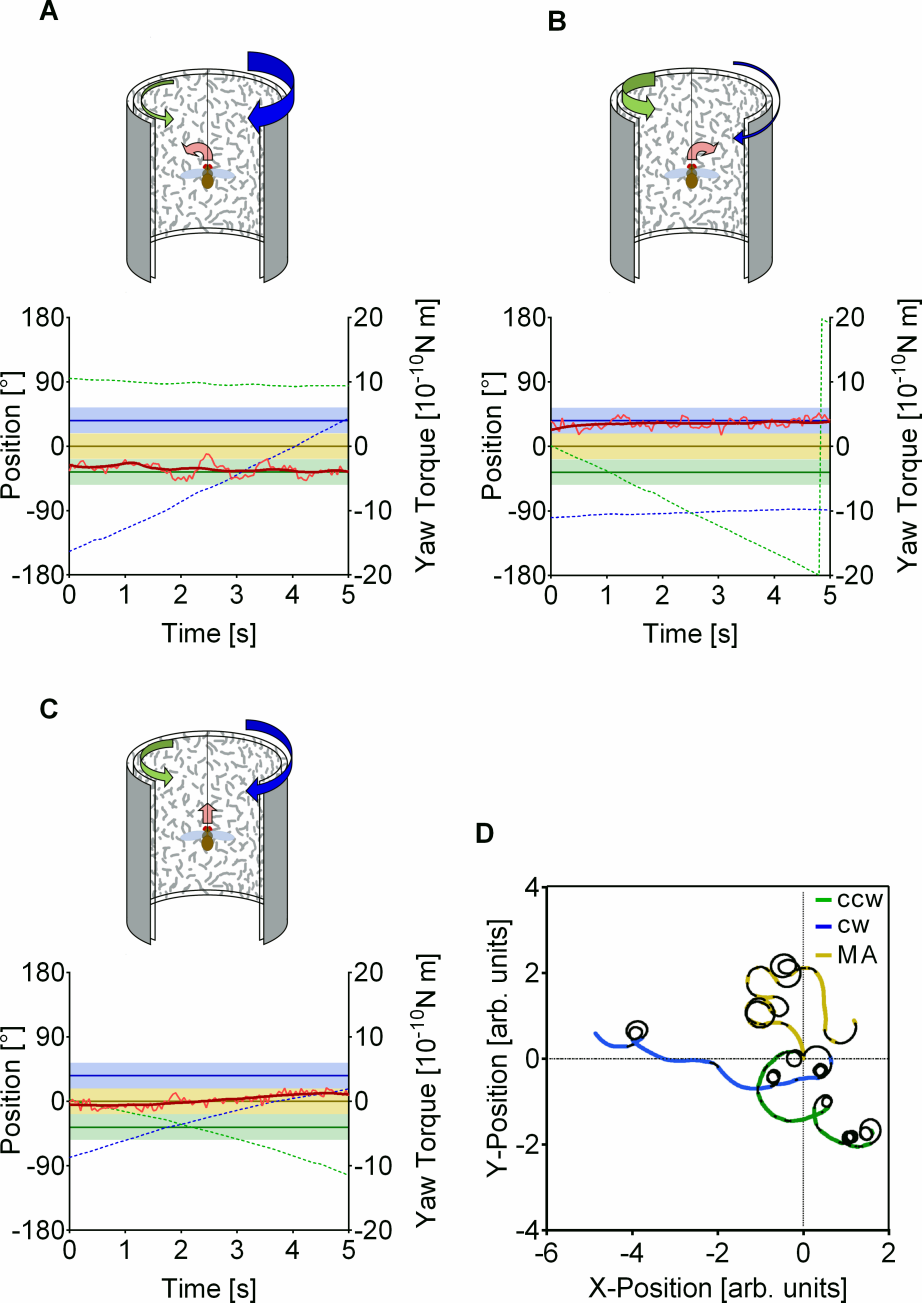
Flight simulator setup and the three flight control behaviors in the TPMP with a pattern contrast of 37%. (A), Example of fly showing SPS_ccw_. Yaw torque (light red, red is moving average) in the green area (solid green line at T = −4 × 1^−10^ Nm shows stabilization value) compensated ccw bias and led to almost stationary pattern orientation (dotted green line). (B), Same as in (A) but for cw bias. Pattern was stabilized with yaw torque in blue area (SPS_cw_). (C), With yaw torque in yellow area around T = 0 Nm (solid black line), the fly stabilized the mean of the two bias values (MA behavior). (D), Virtual flight trajectories of a single 3 min flight in the TPMP in relation to the three references for straight flight, the two patterns (green, blue) and the MA (yellow), assuming a constant flight velocity (i.e., constant thrust). Underlying data can be found in S1 Data. arb, arbitrary; ccw, counterclockwise; cw, clockwise; MA, motion average; SPS_ccw_, counterclockwise single pattern stabilization; SPS_cw_, clockwise single pattern stabilization; TPMP, transparent panorama motion paradigm.

So far, the motion stimulus was unambiguous. To provide ambiguity, we added a second transparent random dots pattern covering the entire arena. In both patterns, the individual pattern elements were opaque, so no change in local contrast occurred within the pattern elements when two of them overlapped. The two textures were identical, however, one was flipped vertically to prevent a complete overlap. If both were linked to the fly's yaw torque in a closed loop with the same coupling parameters, they moved coherently. To generate the ambiguity in the TPMP, we added a rotatory bias to each of the textures, a clockwise (cw) bias to one and a counterclockwise (ccw) bias to the other. This generated a relative angular motion of constant velocity between the two patterns, independent of the fly's yaw torque, while their velocities still depended upon the fly's yaw torque as well. The fly could stabilize each of the patterns but only one at a time. For this, it had to let the other pattern rotate without responding to that motion component. Now the fly had the options to generate optomotor balance with one or the other texture (Fig 1A and 1B). This is what happened: The fly tied all the coherently moving motion elements of the entire visual field together and treated the two motion components as separate entities generating optomotor balance with one or the other pattern (Fig 1A and 1B). We called the stabilization of one of the components single pattern stabilization (SPS) and distinguished the two behaviors according to the bias stabilized (cw SPS [SPS_cw_] and ccw SPS [SPS_ccw_]).

SPS was not the only type of orientation behavior the fly generated. It could also use the vector sum of the two bias components as a reference for straight flight. We called this motion average (MA) behavior (Fig 1C). Further inspection of the yaw torque traces showed that the fly had various strategies at its disposition for stabilizing the motion components and the MA. Examples are shown in S1 Fig. The fly would, for instance, generate fast, large yaw torque modulations around the value that would stabilize one motion component (S1D Fig). Or it would keep its yaw torque level at the zero baseline and suppress net rotation of one of the patterns by a sequence of saccades towards that side (S1B and S1C Fig). Also, with MA behavior, different strategies could be observed (Fig 1C; S1E Fig). This is not a special feature of the TPMP, as different strategies for optomotor balance could also be observed with unambiguous stimuli (S2 Fig).

**Fig 2:**
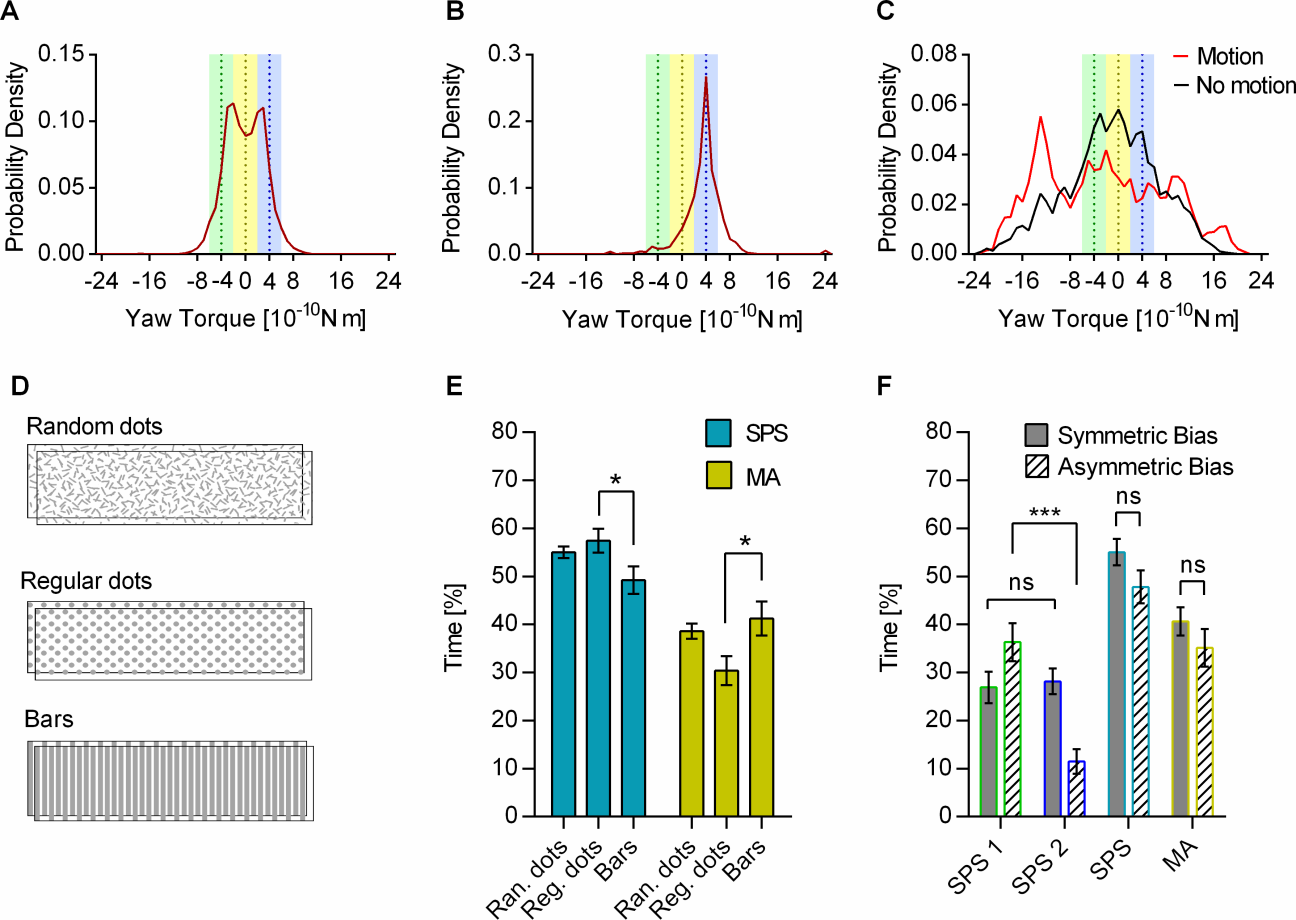
Evaluation, patterns, and bias settings used in the TPMP. (A), SPS led to bimodal distribution of MA of yaw torque. Mean histogram of MA of yaw torque over 2 s with random dots patterns at 37% pattern contrast. Colored areas indicate the ranges where cw (blue) and ccw (green) SPS as well as MA behavior (yellow) were detected; the dotted lines indicate the respective exact stabilization and MA values. (*n* = 20 flies). (B), Stabilization of a single random dots pattern with the second one stationary at the same contrast led to unimodal yaw torque distribution with the peak at the stabilization value. Mean histogram of moving average over 2 s (*n* = 20 flies). (C), With the feedback in the TPMP switched off (open loop), flies showed a broader, multi-modal yaw torque distribution (red; cw: 20° per s, ccw: 20° per s) than in open loop without any motion stimuli (black; cw: 0° per s, ccw: 0° per s), but with the patterns still present (*n* = 11 flies). (D), Overlays of the different patterns used in the TPMP. (E), Different panorama patterns gave similar results. Only between Reg. dots and bars were significant differences found (mean ± SEM; *n* = 20 flies per group, F_SPS_[F(2,57) = 3.341, *p* = 0.0425, R^2^ = 0.1049, ANOVA] with Tukey’s multiple comparisons test [*p* = 0.0384], F_MA_[F(2,57) = 3.988, *p* = 0.0239, R^2^ = 0.1227, ANOVA] with Tukey’s multiple comparisons test [*p* = 0.0238]). (F), Asymmetric bias settings caused preferential SPS with the less biased pattern. Flies performing in the TPMP with one bias set to 0° per s (SPS 1 hatched) and the other set to 40° per s (SPS 2 hatched) showed a significant preference for the pattern without a bias (*t*(19) = 4.340, *p* = 0.0004, paired *t* test). SPS 1 and SPS 2 values with a symmetric bias of 20° per s (solid) were not different (*t*(19) = 0.2384, *p* = 0.8142, paired *t* test). Also, for overall SPS (*t*(38) = 1.654, *p* = 0.1063, *t* test) and MA behavior (*t*(38) = 1.117, *p* = 0.2708, *t* test), values did not differ significantly between the two bias settings (*n* = 20 flies per group). Underlying data can be found in S1 Data and https://doi.org/10.6084/m9.figshare.5786922.v1. ccw, counterclockwise; cw, clockwise; MA, motion average; ns, not significant; Ran, random; Reg., regular; SPS, single pattern stabilization; TPMP, transparent panorama motion paradigm.

As stated in the introduction, we took it that the three modes of pattern stabilization behavior (SPS_cw_, SPS_ccw_, and MA) reflected the flies’ responses to the three possible references for straight flight within the transparent motion stimulus. To calculate the duration and frequency of these modes, we classified them as follows: to be scored as ccw bias stabilization, yaw torque had to be between −2 × 10^−10^ and −6 × 10^−10^ Nm (green colored domain in Figs 1 and 2). To go as cw bias stabilization, yaw torque had to be in the blue domain (2 × 10^−10^ to 6 × 10^−10^ Nm), and it was taken as MA behavior in the range between −2 × 10^−10^ and 2 × 10^−10^ Nm (yellow domain). As the coupling coefficient between pattern motion and yaw torque in the flight simulator was very low compared to freely rotating flies [20], and very short yaw torque fluctuations therefore had little influence on pattern motion, the moving average over 2 s of the yaw torque (dark red traces in Fig 1A–1C) was used for scoring as it reflects the angular velocity of the patterns better than the nonaveraged yaw torque values. The low coupling coefficient was chosen to maximize the difference between the SPS values while keeping the relative angular velocity of the two biases low. The TPMP also worked with a higher coupling coefficient (S3 Fig). The optomotor responses to a single random dots pattern at 37% contrast at different angular velocities showed that there was no difference in the strength of the motion stimulus in the range in which a nonstabilized pattern usually moved in the TPMP (S4 Fig).

The crucial property of the flies’ behavior in the TPMP, which allowed us to classify it as multi-stable, was its continuous alternation between the three stabilization modes. In Fig 1D, this property is visualized for a single 3-min experiment. For each of the three possible references for straight flight, an artificial position trace was calculated, assuming a constant forward velocity. The colored sections indicate where the respective behaviors were scored regarding the yaw torque domains in Fig 1 and Fig 2A–2C. As expected from the classification, this mostly applied to stretches of no or low curvature.

The experiment in Fig 2A shows that SPS_cw_ and SPS_ccw_ were the predominant behaviors in the TPMP with random dots patterns and 37% pattern contrast. The flies preferably responded to the single motion components instead of the compound motion as is apparent from the bimodal distribution of the yaw torque.

The peaks, however, are not at the exact SPS values, but shifted about 12.5% towards the other SPS value. As the yaw torque distribution of the stabilization of a single pattern without incoherent motion (Fig 2B) showed no peak shift, it cannot be a result of the rotatory bias. Hence, we hypothesized the peak shift in the TPMP to result from a residual response to one of the references not used, an issue we address further in a later part of this article.

In S1 Fig, examples for the different strategies (yaw torque patterns) for SPS and MA behavior are described. A salient difference between these yaw torque patterns and the ones shown in Fig 1A–1C is the average amplitude of the yaw torque modulations. Because more of the strategies for SPS required stronger yaw torque fluctuations, we wanted to find out whether the individual mean yaw torque amplitude influenced the scoring of SPS and MA behavior. For each fly, the mean yaw torque amplitude was calculated and compared to the amount of SPS and MA behavior. No correlations were found (S5 Fig). This suggests that the scoring of the different behaviors was independent of the yaw torque modulations.

As Fig 2C shows, the component selectivity the flies expressed appeared not to depend on the feedback provided in the TPMP. With the same incoherent wide-field motion stimulus as in the closed-loop TPMP, the yaw torque distribution was much wider and possibly trimodal, compared to the distribution without any motion when the feedback was switched off. In the latter case, the distribution was unimodal and centered around zero. Obviously, the respective yaw torque ranges at which SPS and MA behavior were detected in the closed-loop TPMP held no meaning for the flies in the open-loop TPMP, which would have made the classification of these behaviors hard in open loop.

The continuously alternating choice behavior in the TPMP did not require random dots textures. We also used 20 evenly spaced vertical stripes or regular dots (Fig 2D). The results were similar, although we found a significant difference between the regular dots and the vertical stripes (Fig 2E). With the latter two patterns, the elements completely overlapped every 0.45 s because of the bias condition, which resulted in a whole-field flicker. This did, however, not seem to have an influence on the overall behavioral choice of the flies. Because the basic horizontal pattern wavelength of the regular dots and the bar pattern was the same, some other pattern feature had to be the cause of the difference in SPS and MA behavior between the two.

Interestingly, we found that the flies also showed a response to the individual components of the transparent motion stimulus with the regular dots patterns and without feedback (S6 Fig). For the random dots patterns, as described above, this was not as surprising, as this is also the case in humans [15]. But locally balanced transparent motion stimuli, like the regular dots pattern used here, are not perceived as transparent by human subjects. Although the response to the individual motion components in the open-loop TPMP appeared to be slightly smaller with regular than with random dots patterns, the yaw torque distribution was still clearly multi-modal, which was not the case without the transparent motion stimulus. Because *Drosophila* shows no behavioral response to a whole-field flicker [21], we considered this effect to be a response to the transparent motion stimulus.

When the same relative rotatory bias between two patterns was injected into the feedback loop, but all of it was added to the closed-loop motion of one of the patterns, the flies stabilized the unbiased pattern significantly more often than the biased one (Fig 2F), which required less yaw torque for SPS. After all, equal forces on both wings, which are in concordance with the internal zero-torque reference of the fly [20], are more likely to generate straight flight and, presumably, are less demanding than strong turning behavior.

### Pattern contrast has an unexpected effect on behavior in the TPMP

**Fig 3:**
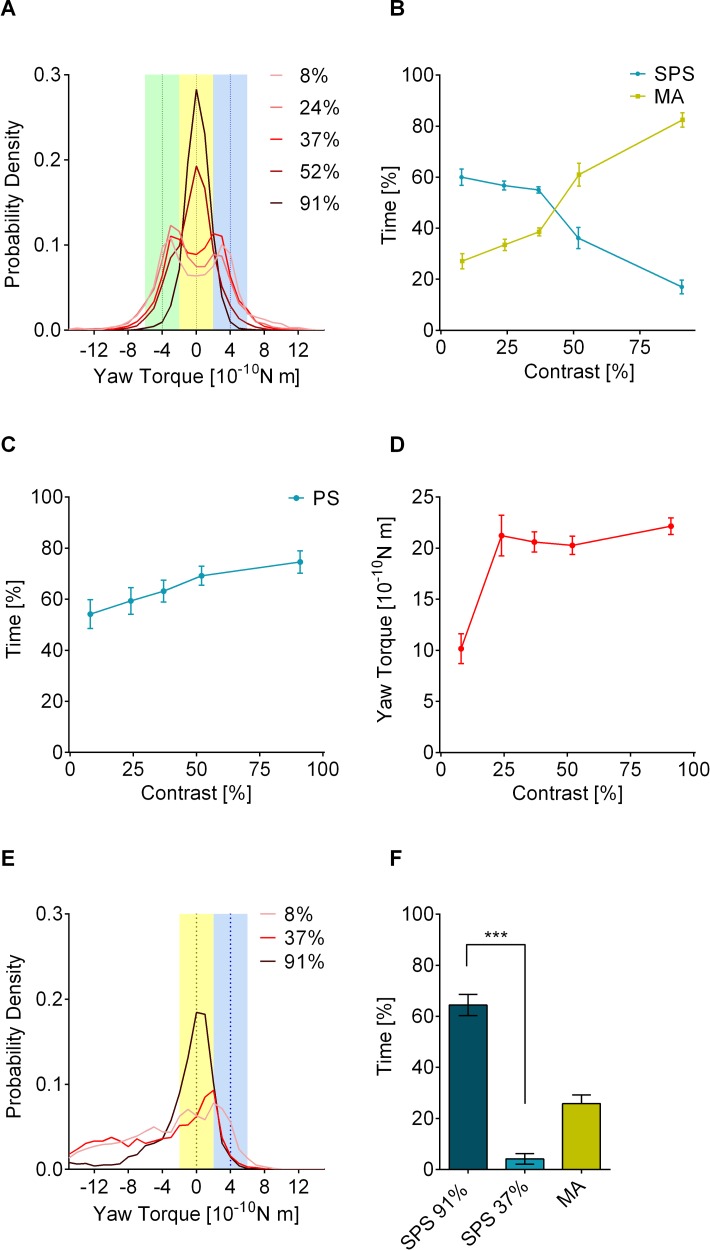
Pattern contrast dependence of SPS and MA. (A), Mean histograms of yaw torque (moving average over 2 s) in the TPMP with random dots patterns (see Fig 2A) under various contrast conditions. High pattern contrast led to unimodal distribution, whereas low contrast resulted in bimodal distribution. Blue and green dotted lines indicate the exact stabilization values for single patterns; the black dotted line for MA behavior; blue and green areas indicate the yaw torque ranges in which SPS is scored; the yellow area where MA is scored. (mean; *n* = 20 flies per contrast condition). (B), Low pattern contrast increased SPS (sum of SPS of cw and ccw bias; mean ± SEM; *n* = 20 flies per contrast condition), whereas high contrast led to MA behavior. (C), Stabilization of a single pattern gradually increased from 8% to 91% contrast. Pattern with a rotatory bias of 20° per s cw or ccw with the second pattern stationary. (mean ± SEM; *n* = 20 flies per contrast condition.) (D), Contrast dependence of the optomotor response (for experimental details see Materials and methods). It differed from that of SPS as well as that of MA behavior. (mean ± SEM, *n* = 36 flies per contrast condition). (E), OL motion increased SPS peak shift. Yaw torque distribution (2 s moving average) with one random dot pattern in CL and another one moving with 20° per s at three contrast values between 8% and 91% (mean, *n* = 20 per contrast condition). For all contrast conditions, the distributions are skewed into the direction of the OL motion. SPS was scored in the blue, MA behavior in the yellow area. (F), Asymmetric contrast settings caused preferential SPS with the higher contrasted pattern. Flies tested in the TPMP with one random dots pattern at 91% contrast and the other at 37% contrast showed a highly significant preference for the 91% contrast pattern. MA behavior was slightly below the level of MA behavior with two 37% contrast patterns (mean ± SEM; *n* = 28 flies; W = −390, *p* < 0.0001, Wilcoxon matched-pairs signed rank test). Underlying data can be found in S1 Data, https://doi.org/10.6084/m9.figshare.4668400.v1 and https://doi.org/10.6084/m9.figshare.5786931.v1. ccw, counterclockwise; CL, closed loop; cw, clockwise; MA, motion average; OL, open loop; SPS, single pattern stabilization; TPMP, transparent panorama motion paradigm.

As already mentioned above, we found the that time the flies spent with SPS in the TPMP was strongly dependent upon pattern contrast. The random dots patterns of Fig 2D were used. At the lowest contrast measured (8%), the flies spent about 60% of the time with SPS and less than 30% with MA behavior (Fig 3). At an intermediate contrast of 37%, which was also used in the experiments in Fig 1, SPS was only slightly lower and MA behavior only slightly higher than at 8% contrast. At the highest contrast (91%), the flies predominantly generated MA behavior (82%) and only rarely generated SPS (17%), with individual flies occasionally displaying constant, very stable MA behavior (S7 Fig). Altogether, in the TPMP, (Fig 3A and 3B) SPS decreased with increasing contrast.

For comparison, we tested stabilization with a single random dots pattern in the closed loop; the second one stationary (Fig 3C). Flies showed reliable stabilization of a single pattern for contrast values between 8% and 91%, with stabilization increasing with contrast. We also tested a pattern contrast of 4%, but only found a very low pattern stabilization (16.89 ± 2.52, *n* = 20 flies) compared to 8% contrast (t(38) = 6.029, *p* < 0.0001, *t* test), which is why the flies were not tested at contrast levels lower than 8% in the TPMP.

We also measured the basic optomotor response (Fig 3D). As with the single pattern in closed loop (Fig 3C), it did not reflect the contrast dependence of SPS in the TPMP. Here, the optomotor response remained stable over all contrast values measured in the TPMP, except the 8% contrast condition, at which the optomotor response of the flies was significantly lower. In that contrast range, however, we observed no significant changes in the TPMP. The inverted contrast dependence of SPS was only found in the TPMP and could also be observed with the regular dots patterns (S8 Fig). We hypothesized that it might be due to a rivalry between SPS and MA.

Interestingly, the peak shifts in the yaw torque histogram at intermediate contrast (37%) described above (Fig 2A) were not present at low contrast (8%) with regular dots patterns (S8A Fig) and only very weak with random dots patterns (Fig 3A). If, as hypothesized above, the peak shift was indeed a residual response to the nonstabilized pattern during SPS, this would make sense: at low contrast, the motion response to the patterns was weaker than at intermediate contrast (Fig 3D, S8D Fig), therefore the response to the nonstabilized pattern in the TPMP at low contrast might also have been lower or more easily suppressed.

To test this hypothesis further, we switched off the feedback for one of the two patterns (Fig 3E). Now, the flies could only stabilize one pattern while suppressing their motion response to an open-loop motion component. They could also do MA behavior by integrating the closed- and the open-loop motion component, thereby also reaching the low net rotation that defines MA behavior. Additionally, they could follow the open-loop motion with an optomotor response, not actively controlling any part of the visual stimulus. Because one of the two patterns was not coupled to the flies’ yaw torque anymore, the relative angular velocity of the two patterns was no longer constant. By inducing this feedback asymmetry, we intended to find out whether this had any influence on the peak shift of SPS or on MA behavior. Fig 3F shows that, for SPS, the peak shifts were more pronounced than with both patterns in closed loop for low and intermediate contrast, whereas no peak shift was observed for the MA behavior at high contrast. As SPS was the prevalent behavior at low and intermediate contrast in the regular TPMP (Fig 3A and 3B) and MA behavior at high contrast, we conclude that this was also the case in the altered feedback situation. At all contrast values measured here, it must also be considered that at very high yaw torque values as they were measured here, resulting from the optomotor response to the open-loop motion, the closed-loop pattern rotated very fast, which may have led to a decreased motion stimulus of that pattern [18] and therefore to a decrease of its stabilization at low and intermediate contrast. At high contrast, this possible difference in the stimulus strength between the two motion components may have led to a decrease in MA behavior. In any case, by inducing feedback asymmetry in the TPMP, we could confirm that the SPS peak shift observed in the TPMP at intermediate contrast is a result of the motion of the nonstabilized pattern and not of the compound motion of the two motion components.

When the two patterns differed in pattern contrast, the flies spent significantly more time stabilizing the high-contrast texture and less time with the low-contrast texture (Fig 3F). A similar effect has been observed when flies were given the choice between differently contrasted vertical bars, where they predominantly chose the higher contrasted one [22].

### Pattern element distance influences behavior in TPMP

**Fig 4:**
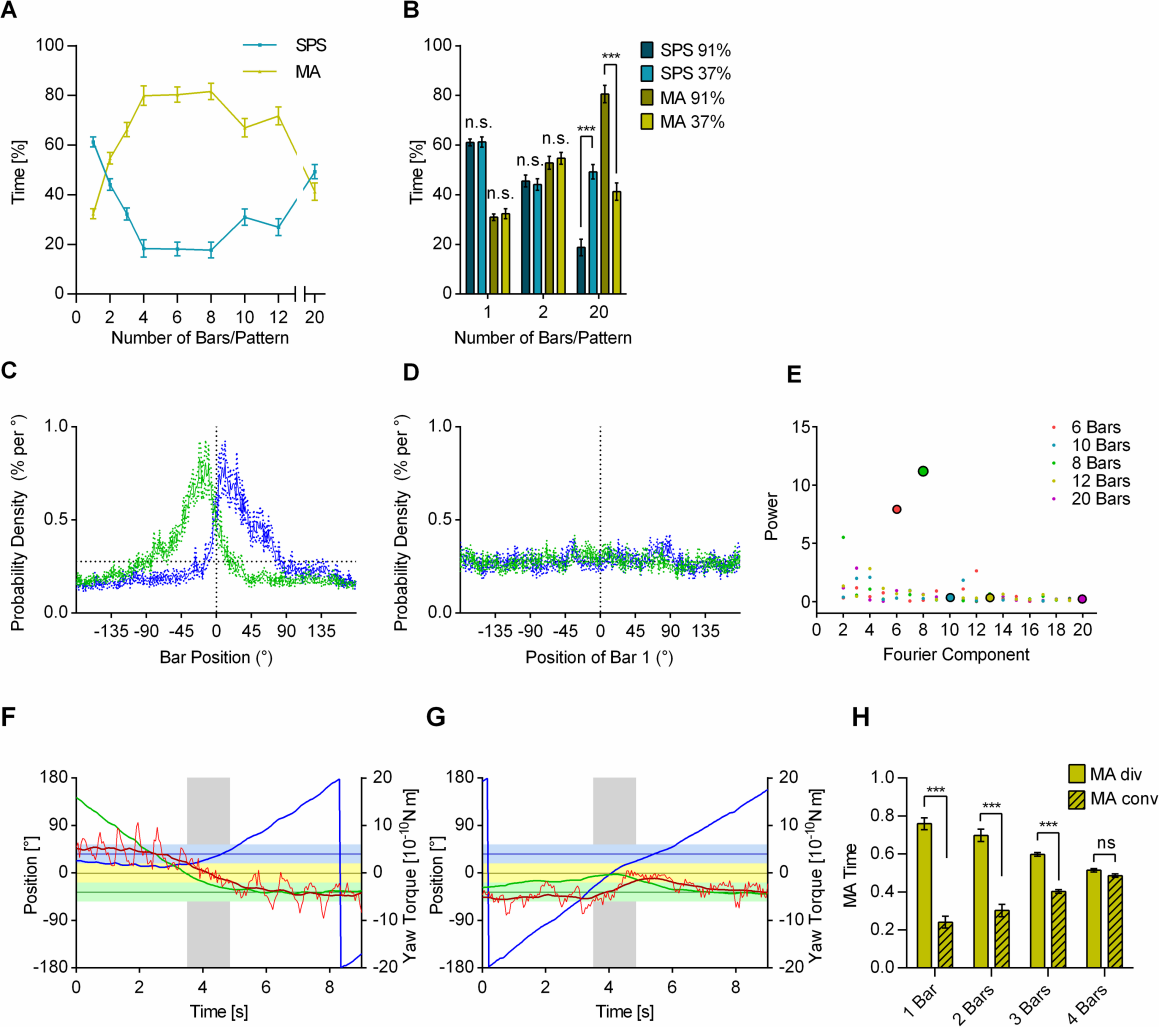
Object response may contribute to MA behavior. (A), With a low number of bars, SPS decreased when their number increased, and with many bars SPS went up again with increasing numbers. MA behavior developed inversely (mean ± SEM; *n* = 20 flies per number of bars). (B), Increased contrast had no effect on ratio of SPS and MA behavior with low number of bars, but with 20 bars the effect was highly significant (mean ± SEM; *n* = 20 flies per number of bars and contrast condition; data of the 37% contrast condition are the same as in (A); SPS 1 Bar: t(38) = 0.0686, *p* = 0.946, *t* test; MA 1 Bar: U = 188.5, *p* = 0.764, Mann-Whitney test; SPS 2 Bars: t(38) = 0.4210, *p* = 0.676, *t* test; MA 2 Bars: t(38) = 0.521, p = 0.606, t-test; SPS 20 Bars: U = 26, *p* < 0.0001, Mann-Whitney test; MA 20 Bars: U = 15, *p* < 0.0001, Mann-Whitney test). (C), With one vertical bar per pattern (width = 6°; contrast: 37%), the flies preferentially stabilized the bars in the frontal visual field on the side where their bias drove them progressively. Position histograms of the one bar per pattern experiment of (A). Green: bias ccw; blue: bias cw. Horizontal dotted line indicates chance level. (D), With 20 evenly spaced bars per pattern, no 18° modulation of the position histograms is apparent. Horizontal dotted line: chance value as in (C). (E), Power spectra of position histograms of orientation in closed loop with a single pattern of 6, 8, 10, 12, or 20 vertical bars (*n* = 20 flies per number of bars). Fourier transform showed fixation of bars for the 6- and 8-bar patterns but not for those with 10, 12, and 20 bars. The color code indicates the number of bars in the respective experiment. (F), A selected 9 s flight episode with one bar per pattern, in which the fly switched from fixating the cw bar to fixating the ccw bar after the bars cross each other. Grey area indicates the time during which average yaw torque (light red) was in the MA range. (Compare to Fig 2). (G), A selected 9 s flight episode in which the fly stabilized the ccw bar shortly interrupted this behavior in favor of MA behavior (grey area) after the bars cross, then returned to stabilizing the ccw bar. (H) Flies in the 1–3 bar/pattern experiment in (A) showed significantly more MA behavior with diverging bars. Bar chart of the fraction of time of MA behavior when the two bars div and conv (1 bar: t(19) = 5.082, *p* < 0.0001, ratio paired *t* test; 2 bars: t(19) = 5.177, *p* < 0.0001, ratio paired *t* test; 3 bars: t(19) = 10.14, *p* < 0.0001, ratio paired *t* test; 4 bars: W = −68, *p* = 0.2162; Wilcoxon matched-pairs signed rank tests). Underlying data can be found in S1 Data, https://doi.org/10.6084/m9.figshare.4668559.v1 and https://doi.org/10.6084/m9.figshare.4668565.v1. ccw, counterclockwise; cw, clockwise; conv, converging; div, diverging; MA, motion average; SPS, single pattern stabilization.

In the TPMP with varying contrast values, we observed that higher contrast values led to increased MA behavior. With an increase in contrast, the single pattern elements increased in salience. Another way to vary the salience of single pattern elements is to vary pattern element density. To examine this closer, we switched from textures of randomly or evenly distributed dots to evenly spaced vertical bars (Fig 2D) and varied their number in the arena (Fig 4A).

With 20 bars in each panorama, the behavior of the flies resembled the one observed with the dot textures. The SPS at high pattern contrast (91%) was very low, even slightly lower than with the random dots patterns. MA behavior was abundant. At 37% contrast, the two behaviors occurred about equally often (Fig 4B).

With a single bar per pattern, the flies were confronted with a profoundly different stimulus situation, two moving objects, neither of which was likely to reflect self-rotation. One might have expected to find only MA behavior. However, as is well known, flies tend to fixate isolated landmarks [19,23]. What we found is that the flies stabilized one bar in the frontal visual field on the side where its bias would move it progressively (Fig 4C). With the data evaluation used in the present study, we found about the same amount of SPS and MA behavior as with the dot patterns (Fig 3B, Fig 4A). Significantly, however, no inverted contrast dependence was observed for SPS with one or two bars per pattern (Fig 4B).

With two bars, the time the flies had to fixate one bar before a second bar entered the frontal visual field was correspondingly shorter. Hence, with an increasing number of bars, SPS strongly decreased (Fig 4A). This reached a low plateau stretching from 4–8 bars per pattern, before SPS increased again with 10 bars and more. With 20 bars, no peaks in the position histogram indicated object fixation (Fig 4D). With a single panorama, object fixation could be observed for up to eight evenly distributed bars, as the experiment of Fig 4E shows. There, Fourier transforms were performed on the cumulated position histograms of 20 flies, stabilizing a single pattern with a certain number of evenly spaced bars. As the fixation of individual bars resulted in peaks at the respective points in the position histograms, a Fourier transform would show peaks at the Fourier component corresponding with the number of bars in the pattern. Interestingly, the absence of peaks in the position histogram of a single pattern coincided with the re-increase of SPS in the TPMP (10 bars per pattern). Taking all these observations together suggests that with few isolated bars and, alternatively, with many bars or textures, SPS was mediated by different mechanisms.

Turning to MA behavior, we found a different situation. As Fig 4A shows, MA behavior was most prominent with 4–8 bars per pattern. But short phases of MA behavior could also be observed with only a single bar per pattern, where they interrupted SPS right after the two bars had crossed and were now diverging (Fig 4F and 4G). Altogether, MA behavior with few (1–3) bars occurred significantly more often when the bars were diverging than when they were converging (Fig 4H), independently of where in the visual field they were previously fixated, although the fact that the bars were predominantly fixated on the side where their bias moved them progressively meant that MA usually occurred when both bars were diverging on the same side of the visual field. As the number of bar crossings increased with the number of bars per pattern, this explained the increase of MA behavior with an increasing number of bars as was found with 1–4 bars (Fig 4A). With more than 8 bars per pattern, however, MA behavior decreased again, suggesting that now the flies no longer responded to the bars as separate objects but rather as the coherently moving elements of larger entities, therefore generating other behaviors but object fixation.

### Temporal dynamics of switching suggests endogenous activation

One of the well-examined aspects of multi-stable perception in humans is the temporal dynamics of the perceptual alternations. Levelt [24] described that in humans the distribution of percept durations has a distinct right-skewed unimodal shape following a gamma distribution, as was later also found in other studies [25–28]. We wanted to know whether the switching behavior of *Drosophila* in the TPMP resembled multi-stable perception paradigms in other animals and humans and thus examined its temporal dynamics.

One of the characteristic properties of the dynamics in higher animals and humans is its stability over time [25,29]. To measure this in flies, we extended the experiment to 6 min and evaluated the trend of SPS over this period. As we wanted to compare the dynamics to bi-stable perception in humans, we used a pattern contrast of 8%. Under that condition, MA behavior was the least frequent. Yaw torque distributions of individual flies performing in this experiment proved to be multi-modal (Hartigans dip test; S1 Table), except for one fly that expressed MA behavior most of the time. All other flies alternated between the different interpretations of the stimulus in the TPMP, particularly between SPS_cw_ and SPS_ccw_.

**Fig 5:**
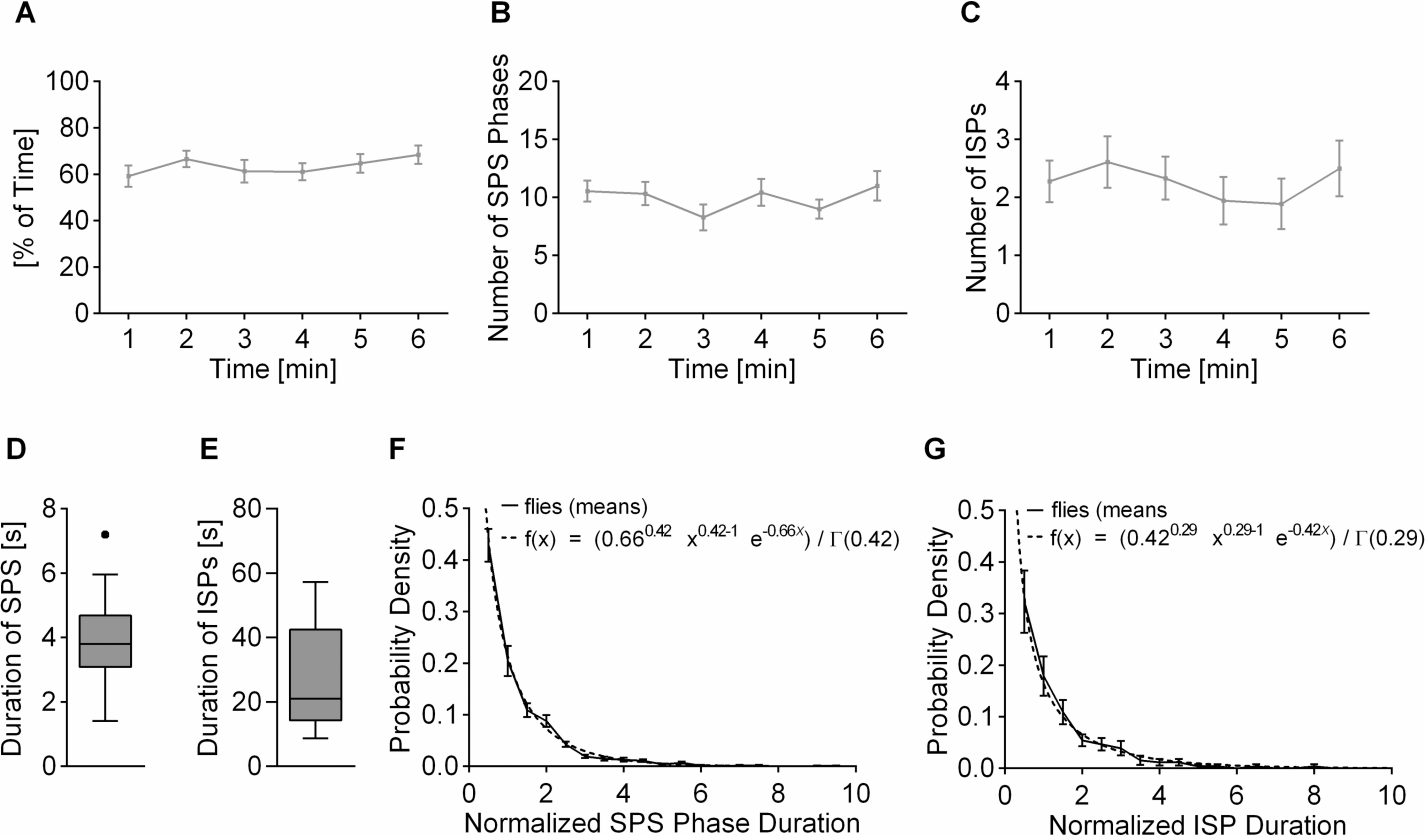
Temporal dynamics and behavioral stability over time in the TPMP. (A), SPS did not change over time. Mean SPS values per minute measured with random dots patterns at 8% contrast (mean ± SEM; *n* = 18 flies; F(3.84, 65.21) = 0.94, *p* = 0.446, R^2^ = 0.052, rm-ANOVA). (B), Number of SPS phases did not change over time. Mean number of SPS phases per minute (Q(5) = 7.45, *p* = 0.189, Friedman test). (C), Mean number of ISPs per minute. One ISP was detected as onset of SPS 1 when the last SPS was SPS 2 and vice versa (Q(5) = 5.502, *p* = 0.357, Friedman test). (D), Mean duration of SPS phases differed among flies. Duration of one SPS phase was calculated as t(SPS1_offset)-t(SPS1_onset) or t(SPS2_offset)-t(SPS2_onset), respectively. Tukey-Boxplot. (E), Mean duration of ISPs differed strongly among flies. Duration of one ISP was calculated as t(SPS2_onset)-t(SPS1_onset) or t(SPS1_onset)-t(SPS2_onset). Tukey-Boxplot. (F), Probability distribution of normalized SPS phase duration fit gamma distribution (R^2^ = 0.84). Individual SPS durations were normalized to the mean SPS phase duration of the respective fly. A replicates test for lack of fit showed no lack of fit (F = 0.24, *p* = 0.999). (G), Probability distribution of normalized ISP duration fit gamma distribution (R^2^ = 0.55). Single ISP durations were normalized to the mean ISP phase duration of the respective fly. A replicates test for lack of fit showed no lack of fit (F = 0.13, *p* = 1). Underlying data can be found in S1 Data and https://doi.org/10.6084/m9.figshare.4668376.v1. ISP, inter-switch-phase; SPS, single pattern stabilization; TPMP, transparent panorama motion paradigm.

No significant changes were observed for the overall time spent with SPS (Fig 5A) and for the number of SPS phases per minute (Fig 5B). As an alternative to SPS duration, we also recorded the time between the onset of SPS with one pattern to the onset of SPS with the other pattern, which we termed inter-switch-phase (ISP). The frequency of ISPs was also stable over time (Fig 5C). Mean durations of ISPs were much larger and frequencies much lower than those of SPS phases. This was in part due to short interruptions in SPS phases by MA or “other” behavior, which were scored the same as the regular switches between behaviors. The mean SPS phases and ISPs of individual flies showed considerable variation (Fig 5D and 5E), as is also typical for the alternation process in multi-stable perception [25,30–32]. There, the coefficient of variation for the individual mean duration of the percepts typically lies between 0.44 and 0.75 [32]. In the TPMP, the coefficient of variation for the individual mean duration of the ISPs was 0.59, so it was within that range.

As mentioned above, in human multi-stable perception, the distribution of percept durations follows a gamma distribution [25]. Its probability density is given by

$$f(t|k,\lambda )=\frac{1}{\lambda^{k}\Gamma (k)}t^{-k}e^{-\frac{t}{\lambda }}$$

The parameters λ and k determine the scale and shape, respectively, of the distribution. The gamma function Γ(k) represents the continuous extension of (*k* − 1)!.

In our study, for a fit between the SPS phase or ISP durations and the gamma distribution above, we normalized the phase durations of all flies to the mean phase duration of each fly and pooled the data of all flies (Fig 5F and 5G). With the SPS phases, we found a very good optimal fit (R^2^ = 0.84) using the parameters λ = 0.66 and k = 0.42 (for more details see Materials and methods). With the ISPs, the optimal fit with λ = 0.42 and k = 0.29 was not quite as good (R^2^ = 0.55), which could be attributed to the lower frequency of these events. The parameters of the two distributions were very similar, which might indicate that they show the same stochastic process. Unlike typically observed with humans and monkeys but as found in experiments on pigeons [6], our values for k were smaller than 1, reflecting the highest frequencies for the shortest durations. The pigeon study [6] found their data to also fit a single-parameter exponential function, which provides a more parsimonious model and might also be applicable with our data. Nevertheless, in the TPMP the temporal dynamics of both kinds of phase durations seemed to indicate that switches between behaviors were not tightly coupled to, for instance, a regularly occurring external stimulus but were influenced by a presumably endogenous stochastic process.

### Alternating behavior does not require binocular stimulation

**Fig 6:**
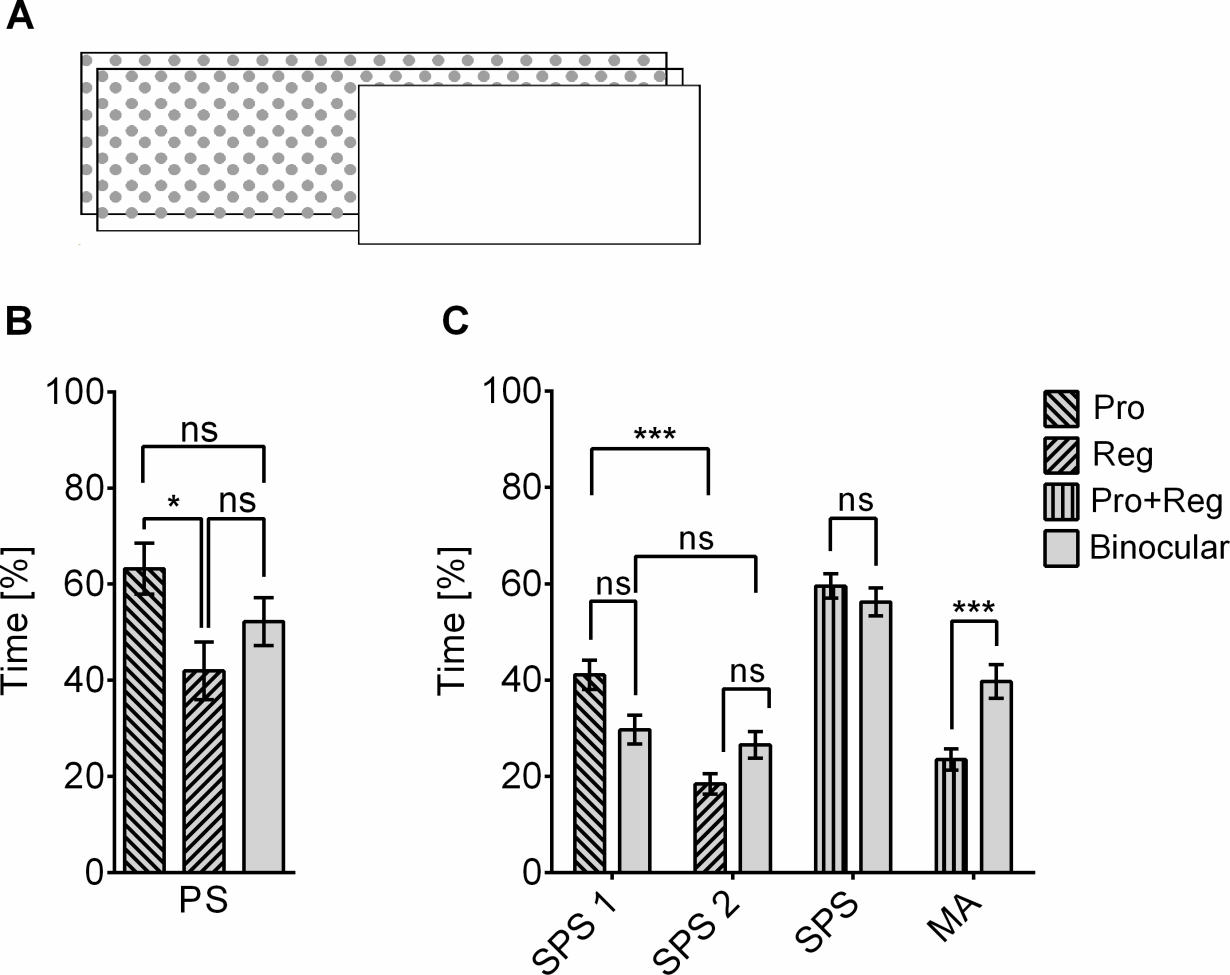
Monocular TPMP reduces SPS for regressively moving bias and MA behavior. (A), Monocular stimulation with regular dots patterns. (B), One pattern in closed loop (progressive or regressive bias, 20° per s), the other stationary (mean ± SEM, *n* = 20 flies per group, F(F(2, 57) = 0.4453, *p* = 0.0272, R^2^ = 0.1188, ANOVA)). Pro bias was more extensively stabilized than Reg bias (*p* = 0.0203, Tukey’s multiple comparisons test). Neither performance value was significantly different from PS with binocular stimulation (grey) (p_reg_ = 0.3822, p_pro_ = 0.3289, Tukey’s multiple comparisons test). (C), In the TPMP, flies with monocular input stabilized the pro moving pattern significantly more than the reg moving one (mean ± SEM; *n* = 21 flies for binocular stimulus, *n* = 42 flies for monocular stimulus; H(3, 123) = 31.3, *p* < 0.0001, Kruskal-Wallis test; p_pro-reg_ = < 0.0001, p_bino1-bino2_ > 0.9999; p_pro-bino1_ = 0.2677, p_reg-bino2_ = 0.3702, Dunn’s multiple comparisons test). With monocular stimulation (pro+reg), MA behavior was reduced compared to binocular stimulation (binocular)(U = 401.5, *p* = 0.57, Mann-Whitney test), while overall SPS was not different (U = 205, *p* = 0.0004, Mann-Whitney test). Underlying data can be found in S1 Data and https://doi.org/10.6084/m9.figshare.4668571.v1. MA, motion average; Pro, progressive; PS, pattern stabilization; Reg, regressive; SPS, single pattern stabilization; TPMP, transparent panorama motion paradigm.

Tang and Juusola [33] have shown that *Drosophila* alternates between cw and ccw motion responses, if these stimulus components are presented simultaneously, but each one only to one half of the visual field, a paradigm reminiscent of binocular rivalry. In the TPMP, both motion components were presented to both visual half-fields. Thus, binocular rivalry would not suppress one of the motion components unless the fly would use only progressive or regressive motion [34] for bias compensation. To test for binocular rivalry, we presented the visual motion only to one eye (Fig 6A). We used the regular dots texture (see above). In a first experiment, we presented only one of the patterns in closed loop, with the second one stationary. The flies were confronted with either a regressive or a progressive bias on the open side of the panorama. Using the classification for SPS above, we found a significantly more abundant stabilization of the progressive bias compared to the regressive one, while neither was different from the stabilization of a binocularly presented pattern (Fig 6B).

Also in the TPMP with the visual input restricted to only one eye, the flies could stabilize both the progressive and the regressive bias (Fig 6C). Overall, SPS was as pronounced as with both eyes. However, the difference in the stabilization of the progressive and regressive bias was highly significant, whereas there was no difference between SPS_cw_ and SPS_ccw_ with both halves of the visual system in operation. To stabilize the regressive bias, the flies had to generate yaw torque towards the side where the visual motion input was blocked. With the progressive bias being visible at the same time, turning to the shielded side seemed to be even less attractive than with only the regressive bias. But although individual flies may have exclusively stabilized the pattern with the progressive bias (S9 Fig), we also found flies that stabilized both patterns about equally often (S10 Fig). Therefore, we concluded that the multi-stable behavior in the TPMP did not depend on binocularity, although the lack of binocularity influenced the choice behavior of the flies. Interestingly, MA behavior was significantly reduced with the monocular stimulation compared to the binocular one, which may lead to the assumption that the zone of binocular overlap could have a special relevance for MA behavior. It could also be at least partially dependent on the equivalence of the two opposing motion stimuli, which is not given in this case, because the stimulus strength of the progressively moving pattern was bigger than the one of the regressively moving pattern when both patterns were moving with the same absolute angular velocity. The fraction of time spent less on MA behavior was shifted to “other” behavior.

## Discussion

Perception is the process and result of the identification, organization, and interpretation of sensory stimuli to represent the environment [35] and provide a basis for directed behavior. Multi-stable perception can occur when a constant ambiguous stimulus allows for several interpretations. With the TPMP, we designed an ambiguous stimulus for *Drosophila* in which the ambiguity is persistent over time but that can still be actively controlled by the fly. The constant ambiguity evoked a multi-stable behavioral response, in which each of the behaviors was coupled to one reference for optomotor balance, i.e., straight flight. Multi-stable phenomena in humans and nonhuman primates are studied as perceptual multi-stability [5, 36–38], which means that they rely on introspection and its report. In *Drosophila*, we measured behaviors that were functionally coupled to one of three references that defined the respective interpretations of the ambiguous stimulus. This raises the question of what the nature of the underlying ambiguity was. As the fly’s primary objective in the flight simulator without an ambiguous stimulus is to stabilize its flight, it can be assumed to also be so in the TPMP. The differing references for optomotor balance provided in the TPMP competed, leading to the multi-stability. Our results indicate that the TPMP produces two ambiguities. One is whether both motion components occur out in the world or one of them signals self-rotation. If only one component is indeed self-rotation, the second ambiguity is relevant: which components are which.

The stimulus ambiguity in the TPMP can also be interpreted in the context of depth perception as either transparent or nontransparent, the latter facilitating MA behavior, because an asymmetry in the feedback alters SPS but not MA behavior (Fig 3E). Also, the ratio between MA behavior and SPS (Fig 2E, Fig 3B, Fig 4A) is influenced by different parameters than the ratio between SPS_cw_ and SPS_ccw_ (Fig 2F). When interpreted as transparent, either the cw- or the ccw-biased pattern can be perceived as the reference for optomotor balance, leading to SPS.

The discovery of multi-stability in fly visual behavior using transparent panoramic motion stimuli reveals some interesting properties of *Drosophila* motion vision and behavior. The perception of transparent motion stimuli can also be multi-stable in humans [10,11], although this has only been shown for two-dimensional transparent motion or plaid motion. A study investigating the processing of plaid motion in blowflies suggested that they express component selectivity in response to the individual moving gratings in a two-dimensional transparent motion stimulus [8], although the physiological findings of a fly equivalent of pattern and component cells in the lobula plate did not fully explain the behavioral phenotype. The component selectivity of one-dimensional transparent wide-field motion stimuli observed in the TPMP in the form of SPS is not predicted by current models of fly motion vision, according to which motion signals of the same strength and opposite direction should cancel each other, if both are distributed evenly over the visual field [7,12,13]. Consequently, only the behavior associated with the integration of the two motion components, MA behavior should be found. In the present study, it can be shown that the visual system of *Drosophila* separates transparent motion stimuli, even if they completely overlap and cover most of the visual field. This does not only occur in closed-loop orientation behavior. Even without visual feedback, the two motion components are received separately and answered by syn-directional yaw torque. Studies of transparent motion vision in humans are usually conducted under gaze fixation, therefore providing much less visual feedback than the TPMP [15,39]. So, the open-loop TPMP provides a better comparison to these experiments than the normal TPMP. Here, an interesting difference between fly and human transparent motion vision is revealed: while in humans, the detection of transparency depends on locally unbalanced motion stimuli as they emerge with random dot patterns, this is not the case in *Drosophila*, because we also observe component selectivity with regular dot patterns—where the local motion is balanced (S6 Fig, S8 Fig). How strongly the component selectivity is expressed in the orientation behavior of the flies in closed loop depends on at least two stimulus parameters, pattern contrast, and pattern element density.

An increase in contrast and a decrease in pattern element density both increase the salience of the single pattern elements in a panorama pattern and thereby strengthen its figure features. Both also increase the relative abundance of MA behavior in the TPMP. With widely spaced vertical bars moving incoherently, MA appears to be a consequence of ipsilaterally diverging figures (Fig 6F–6H). At intermediate contrast, with increasing density of the bars, MA behavior starts to decrease at a bar distance where fixation responses are no longer apparent. Parsimony would suggest that, as both high pattern contrast and high pattern element distance favor MA behavior in the TPMP and MA behavior at low pattern element density is elicited by figure responses, MA behavior is generally a consequence of figure detection and responses. SPS would then be a behavioral response to elementary wide-field motion in the absence of figure detection. This conjecture is supported by the fact that, regardless of the contrast level and the pattern element density, panorama pattern elements with stronger figure features, like vertical bars, which evoke edge detection [40], result in higher MA behavior values than panorama pattern elements with weaker figure features, like vertical rows of dots (Fig 2E, S8B Fig, Fig 4B). Moreover, the contrast effect only occurs when the figure features are not already very high due to a low pattern element density (Fig 4B). It has been observed before that, when given the choice between stabilizing a background motion and fixating a bar, flies choose to fixate the bar [19]. Extrapolating this to the TPMP, the figure motion response resulting in MA behavior suppresses the elementary wide-field motion response, which would result in SPS. The weaker the figure features of the patterns become, the more often SPS is the preferred behavior. Therefore, we conclude that the rivalry between MA behavior and SPS is essentially one between the figure and the wide-field motion systems. It also means that figures can be detected by *Drosophila* on a much smaller spatial scale than previously assumed [20].

Many studies have been dedicated to the difference between figure motion and elementary wide-field motion vision in *Drosophila*, inquiring how the fly discriminates between the two and how it reacts if both are presented simultaneously [19,41–43]. Yet there has only been little research as to how the fly responds to transparent wide-field motion components when they do not differ in their pattern properties. Also, it remains unclear what defines a figure or wide-field motion stimulus as such, given that the motion stimulus of a panorama pattern always must consist of an array of objects, no matter the shape, size, or number of pattern elements. When shown a single, dark, vertical bar, a figure, on a light background in closed loop, the fly will fixate it in the frontal part of its visual field [19]. This behavior is supported by elementary motion vision, but is essentially independent of it [44,45]. With a panorama pattern containing numerous pattern elements, but no salient singularities, the fly will also show stabilization behavior of this pattern [19], independent of the position of a particular pattern element. Lately, several studies have characterized how flies process figure motion [16,41,46], pointing out that usually, moving figures possess both figure and elementary motion features, which are distinguished in the fly visual system. Interestingly, for a certain type of motion-blind flies it can be shown that their most basic motion response, their optomotor reflex to wide-field patterns, is completely abolished at low contrast [44], but a very low, residual response seems to remain at intermediate [44] and high contrast [47]. This response is likely generated by the system processing figure motion [44].

This interpretation of the flies’ behavior does also make sense from an ecological point of view: If a fly interprets the stimulus in the TPMP as an array of objects, the most logical solution to maintain a straight trajectory is to integrate the motion components of the individual pattern elements. If it is interpreted as two panoramic flow-fields, which might both represent a stationary background, it makes more sense to choose a single one as a reference for straight flight.

Previous studies examining a flies’ alternations between different responses to a constant stimulus situation [33,48–50] can ultimately all be explained by spatially selective visual attention, because the competing stimuli were presented in differing parts of the visual field. In human perceptual rivalries, attention is linked to multi-stability, but ultimately independent of it [2]. In the TPMP, the alternations between the two components of the compound visual stimulus in the TPMP are not a consequence of spatially selective visual attention or binocular rivalry, because we can show that they also occur without binocular stimulus presentation (Fig 6). The decrease in MA behavior we observe with monocular stimulus presentation might be a result of the strongly reduced visual input in the frontal part of the visual field of the noncovered eye, as MA behavior appears to be a kind of figure response and the response to figure motion components has been found to be stronger in the frontal part of the visual field [16]. But, as with SPS of the regressively biased pattern, it is not completely abolished, which represents another similarity of the multi-stable behavior of the fly in the TPMP to multi-stability in humans. There, if the stimulus strength and therefore the likelihood for one of the percepts is decreased, so is the time this percept is perceived, but while the ambiguity remains, however weak, the alternations remain [51]. The same can be said for the other parameters, like bias distribution, pattern contrast, and pattern element density, which influenced the choice behavior of the fly in the TPMP. When the likelihood for one of the interpretations of the transparent motion stimulus to be the “correct” one was increased, so was the time the flies spent with the corresponding behavior, and the time with the behaviors corresponding to the “less likely” interpretations was accordingly decreased, but the behaviors corresponding with the less likely interpretations were never fully abolished.

In humans, visual multi-stable phenomena are considered an indication for vision being an active process. This means that the interpretation of a visual stimulus is shaped by the brain as well as by the stimulus, particularly in situations where a stimulus can be interpreted in more than one way [2,52,53]. As already mentioned in the introduction, also in the natural environment of a fly, visual stimuli may not always be unambiguous and its visual system needs a way to derive the optimal interpretation without getting stuck on a potentially wrong one.

The temporal dynamics of the behavioral alternations of *Drosophila* in the TPMP also share most of the characteristics of human multi-stable perception. The stochastic component in the temporal activation pattern of the different behaviors in the TPMP suggests that the behaviors were activated endogenously. So far, this claim has been difficult to prove, but mechanisms providing such stochasticity are now being investigated [54,55].

To summarize, when *Drosophila* in stationary flight is exposed to transparent wide-field motion stimuli, its orientation behavior is multi-stable. This shows that *Drosophila* can process the individual components of a one-dimensional transparent motion stimulus separately and that this kind of stimulus is ambiguous to the fly. To what extent the fly expresses component selectivity depends on several properties of the stimulus, namely pattern contrast and element density. As component selectivity increases with a decrease of the figure features of the stimulus, we conclude it to be the result of wide-field motion vision in the absence of figure detection. The alternations between the different behaviors exhibit a stochasticity reminiscent of the temporal dynamics in human multi-stable perception.

## Materials and methods

### Flies

Flies were cultured at 25°C on standard food medium [56] on a 12 h light/dark cycle with 60% relative humidity. Wild-type flies were of the Wildtype Berlin (WTB) strain. For tethering, 2- to 3-day-old female flies were cold-anesthetized and glued with dental composite (ESPE Sinfony DO3, 3M, Neuss, Germany) to a copper rod (Ø = 0.15 mm, length = 2 mm) using a micromanipulator. The tip of the rod was positioned between the flies’ head and thorax to exclude independent motion of these two body parts. The glue was polymerized with blue LED light (10 s pulse, distance < 5 mm), and the flies were then kept isolated with access to water for 2 to 14 h prior to the experiment.

### Setup

Visual stimuli were presented in a cylindrical arena via fiber optics, and 32 x 180 lightguides connected the inner surface of the arena (Ø = 90 mm, h = 90 mm) with the rectangular frontplate. The arena covered 360° x +/−45° of the flies’ visual field. Computer-generated visual stimuli were displayed on a screen placed directly onto the frontplate. The visual stimuli were controlled by custom-made software written in VB.NET. The rod glued to the fly was positioned in the tip of a syringe, which was then attached to the torque meter and centered in the arena. In the flight simulator, the torque meter transduced yaw torque into an electrical voltage, which was read by a computer by using a data acquisition device (USB-1208 FS; Measurement Computing, Germany). The angular motion of the visual panorama was calculated online from the yaw torque signal and then displayed in real time to the fly.

### Stimulus conditions

Contrast values of the used patterns ranged between 4% and 91%. The RGB values of the white background input were always the same; however, the scattered radiation from the darker pattern elements, or lack thereof, resulted in illuminance values between 42 and 45 lux for the background. Luminance values of the darker pattern elements ranged between 2 lux and 40 lux, resulting in contrast values between 91% and 4%, measured as (E_v(max)_-E_v(min)_)/ (E_v(max)_ + E_v(min)_). Random dots patterns (Fig 2D, adapted from Letratone Sheet LT131 [Letraset]), regular dots patterns (diameter, d = 7°, 20 columns, 6 horizontal rows) or evenly spaced vertical stripes (width, w = 6°) were used as visual stimuli.

For closed-loop experiments, the coupling coefficient was set to −5 (i.e., a yaw torque of 1 × 10^−10^ Nm), and resulted in an angular displacement of 5° against the direction of yaw torque. Unless otherwise stated, the rotatory bias used in the closed-loop experiments was set to ±20° per s. For the optomotor balance controls with just one pattern in closed loop the sign of the rotatory bias was randomly set to either positive or negative for each fly. In the incoherent motion paradigm, one bias value was set to 20° per s, the other to −20° per s. Unless otherwise stated, the experiment duration for the closed-loop experiments was set to 3 min. For the monocular condition, the stimulus input to one eye was eliminated by positioning a white virtual screen covering the arena from −180° to 20° or from −20° to 180°, respectively, over the panorama stimuli. The 20° on the noncovered side accounted for the 15° binocular overlap per side, plus a 5° error margin for positioning the fly in the arena to guarantee exclusive stimulus presentation to one eye.

The optomotor response was tested by rotating one pattern around the fly with a second one stationary to provide the same contrast conditions as in the closed-loop experiments. The pattern was alternately rotated cw and ccw for 9 times for 15 s. The optomotor response was tested at three different angular velocities, 20° per s, 40° per s, and 60° per s.

In the open-loop TPMP, the same settings were used as in the normal, closed-loop TPMP, but the feedback was switched off.

Flies were tested under up to five stimulus conditions, provided they did not stop flying throughout the trial. If they were tested under more than one condition, the order of the stimulus conditions was randomized. If a fly stopped flying for more than three times throughout a trial, the trial was aborted. For the experiment with monocular visual stimuli, all flies were tested under every stimulus condition.

### Data evaluation

Yaw torque recordings were stored on the measuring PC hard disc with a sampling rate of 20 Hz and evaluated after the experiment with custom-made software written in VB.NET. Controls with an asymmetric bias showed that the flies were prone to prefer the pattern with the lower bias (Fig 2F). Because an out-of-alignment setting of the zero yaw torque value had the same effect as an asymmetric bias, flies that showed a strong bias towards one of the patterns (> 75% of the time on one side) under closed-loop conditions were excluded from the data evaluation. Following this criterion, dependent on pattern contrast, between 5% (91% contrast) and 20% (8% contrast) of the flies had to be excluded from evaluation. For the evaluation of the temporal dynamics experiment, all flies that stopped flying throughout the experiment were excluded from evaluation.

The closed-loop experiments were evaluated by calculating the moving average over 2 s of the yaw torque values. The incidences of the resulting values within the range (< ±2 × 10^−10^ Nm for MA behavior; < -2 × 10^−10^ Nm and > −6 × 10^−10^ Nm or > 2 × 10^−10^ Nm and < 6 × 10^−10^ Nm for SPS, respectively) of a behavioral category were then reported as a percentage of the entire experiment time.

For the evaluation of the optomotor response, the first 15-s period was discarded and the other 8 periods were averaged. As we found no difference in the optomotor response to the different angular velocities measured, they were pooled for further evaluation. The overall optomotor response was reported as the mean yaw torque of the last 5 s of the 15-s periods.

### Statistical analysis

Data were tested for normal distribution using a D’Agostino-Pearson omnibus normality test. When they were normally distributed, a Student *t* test was used to test two groups against each other. When no normal distribution could be assumed, a Mann-Whitney test was used to compare two groups. Comparison of more than two groups was achieved by a one-way ANOVA with Tukey’s multiple comparisons test when the data were normally distributed, and with a Kruskal-Wallis test with Dunn’s test for multiple comparisons when they were not normally distributed. Bonferroni corrections were used for multiple comparisons. The curve fitting was done with the method of least squares estimation. Statistical significance was demonstrated as ***p < 0.001, **p < 0.01, *p < 0.05, and nonsignificant (ns) *p* > 0.05.

## Supporting information

S1 Fig Flies show various strategies for SPS and MA behavior. Besides keeping their yaw torque stable at approximately the values of either SPS or MA (Fig 2A–2C), flies also employ other flight maneuvers to stabilize their virtual self-rotation. Some of this is also scored as SPS or MA behavior. (For explanation of plots see legend to Fig 1.) (A), Saccadic tracking. Baseline yaw torque on the nonstabilized pattern while performing large torque spikes to stabilize the other pattern. (B), Baseline saccadic tracking. The fly keeps the baseline of yaw torque on the MA yaw torque value while performing torque spikes to stabilize one pattern. (C), Intermittent baseline tracking. Baseline of yaw torque on the MA value while the fly performs occasional large torque spikes to stabilize one pattern. (D), Oscillating tracking. Yaw torque oscillations around the stabilization value of one pattern. (E), Oscillating MA. Yaw torque oscillations around the MA value. MA, motion average; SPS, single pattern stabilization.
(TIF)

S2 Fig Flies display several yaw torque patterns for the stabilization of a single pattern (PS). (A), Straight stabilization. Flies keep their yaw torque relatively stable at the stabilization value. (B), Oscillating stabilization. Yaw torque oscillates around the stabilization value. (C), Saccadic stabilization. Flies keep their baseline yaw torque at a level different from the stabilization value but stabilize the pattern with torque spikes. PS, pattern stabilization.
(TIF)

S3 Fig Coupling coefficient and relative angular velocity had no significant impact on SPS and MA behavior. Comparison of SPS and MA behavior values for different combinations of cc and rav. Variation of the cc had no significant influence (n (cc = −5° per s; rav = 40° per s) = 15; n (cc = −11° per s; rav = 88° per s) = 19; n (cc = −11° per s; rav = 40° per s) = 20; SPS: F(F(2, 51) = 5.014, *p* = 0.0103, R^2^ = 0.1643, ANOVA, p(−5° per s; 40°per s vs. −11° per s; 88° per s) = 0.2335, Dunnett’s multiple comparisons test)). cc, coupling coefficient; MA, motion average; rav, relative angular velocity; SPS, single pattern stabilization.
(TIF)

S4 Fig Optomotor response to a random dots pattern with 37% contrast does not differ between 20° per s and 60° per s. (A), Mean optomotor response over 15 s (mean ± SEM, *n* = 12 flies per angular velocity setting, 8 trials per fly, for more details see Materials and methods). As the trials were conducted direct successively and the data of the ccw trials then sign inverted for averaging, the initial yaw torque values for the optomotor response are negative. (B), Average optomotor response over the last 5 s (grey area panel A) does not differ between angular velocity settings (mean ± SEM; F(2,33) = 0.166, *p* = 0.847, ANOVA). ccw, counterclockwise.
(TIF)

S5 Fig No correlation between mean YTAs and single SPS and MA behavior in the TPMP. Mean YTA of individual flies was calculated as the mean difference between every two inflection points in the yaw torque trace. The individual flies’ YTA values did not correlate with the amount of SPS (blue) and MA behavior (yellow) they showed (SPS: r = −0.034, *p* = 0.885, Spearman r; MA: r = −0.401, *p* = 0.08, Spearman r). MA, motion average; SPS, single pattern stabilization; TPMP, transparent panorama motion paradigm; YTA, yaw torque amplitude.
(TIF)

S6 Fig Flies show motion response to transparent motion stimuli in open loop with regular dots pattern (Fig 2D). As with random dots patterns (Fig 2C), flies showed a broader, multi-modal yaw torque distribution (red; cw: 20° per s, ccw: 20° per s) to transparent motion without feedback than in open loop without any motion stimuli (black; cw: 0° per s, ccw: 0° per s), but with the patterns still present (*n* = 16 flies). ccw, counterclockwise; cw, clockwise.
(TIF)

S7 Fig Flight trace of TPMP with random dots patterns at 91% contrast. TPMP, transparent panorama motion paradigm.
(TIF)

S8 Fig TPMP with regular dots patterns (Fig 2A) shows same general results as with random dots patterns. (A), Histograms of yaw torque (moving average over 2 s) under various contrast conditions. High pattern contrast leads to unimodal distribution, while low contrast results in bimodal distribution. Blue and green dotted lines indicate the exact stabilization values for single patterns, the black dotted line for MA behavior, blue and green areas indicate the yaw torque ranges where SPS, is scored, and the yellow area where MA is scored. (mean; *n* = 20 per contrast condition). (B), Low pattern contrast increases SPS (sum of SPS of cw and ccw bias; mean ± SEM; *n* = 20 per contrast condition), while high contrast leads to MA behavior. (C), Stabilization of a single pattern increases from 37% to 91% contrast. Pattern with a rotatory bias of 20° per s cw or ccw, with second pattern stationary (mean ± SEM; *n* = 20 per contrast condition). (D), Contrast dependence of the optomotor response. It differs from that of SPS as well as that of MA behavior (mean ± SEM). ccw, counterclockwise; cw, clockwise; MA, motion average; SPS, single pattern stabilization; TPMP, transparent panorama motion paradigm.
(TIF)

S9 Fig Flight trace of TPMP with monocular stimulation on the right eye. Individual flies may ignore the regressively biased pattern completely and perform SPS exclusively with the progressively biased pattern. SPS, single pattern stabilization; TPMP, transparent panorama motion paradigm.
(TIF)

S10 Fig Flight trace of TPMP with monocular stimulation on the right eye. Some flies also regard the regressively biased pattern and perform SPS with both patterns. SPS, single pattern stabilization; TPMP, transparent panorama motion paradigm.
(TIF)

S1 Table Results of Hartigan’s dip test for unimodality for individual flies of 6 min TPMP experiment at 8% contrast. *p*-Values smaller than 0.05 indicate significant bimodality, which applied to 17 out of 18 flies. The fly for which no bimodality could be assumed expressed predominantly MA behavior. MA, motion average; TPMP, transparent panorama motion paradigm.
(XLSX)

S1 Data Data underlying figures and supporting information figures. (XLSX)

## Abbreviations

ccw: counterclockwise
conv: converging
cw: clockwise
div: diverging
ISP: inter-switch-phase
MA: motion average
SPS_ccw_: counterclockwise single pattern stabilization
SPS_cw_: clockwise single pattern stabilization
SPS: single pattern stabilization
TPMP: transparent panorama motion paradigm
WTB: Wildtype Berlin

## References

[1] Brascamp JW, Klink PC, Levelt WJ. The 'laws' of binocular rivalry: 50 years of Levelt's propositions. Vision Res. 2015;109(Pt A):20–37. doi: 10.1016/j.visres.2015.02.019 25749677 10.1016/j.visres.2015.02.019

[2] Leopold DA, Logothetis NK. Multi-stable phenomena: changing views in perception. Trends Cogn Sci. 1999;3(7):254–64. doi: 10.1016/S1364-6613(99)01332-7 10377540 10.1016/s1364-6613(99)01332-7

[3] Sterzer P, Kleinschmidt A, Rees G. The neural bases of multi-stable perception. Trends Cogn Sci. 2009;13(7):310–8. doi: 10.1016/j.tics.2009.04.006 19540794 10.1016/j.tics.2009.04.006

[4] Kornmeier J, Bach M. Ambiguous figures—what happens in the brain when perception changes but not the stimulus. Front Hum Neurosci. 2012;6:51 doi: 10.3389/fnhum.2012.00051 22461773 10.3389/fnhum.2012.00051 PMC3309967

[5] Logothetis NK, Schall JD. Neuronal correlates of subjective visual perception. Science. 1989;245(4919):761–3. 2772635 10.1126/science.2772635

[6] Vetter G, Haynes JD, Pfaff S. Evidence for multi-stability in the visual perception of pigeons. Vision Res. 2000;40(16):2177–86. doi: 10.1016/S0042-6989(00)00070-5 10878279 10.1016/s0042-6989(00)00070-5

[7] Mauss AS, Pankova K, Arenz A, Nern A, Rubin GM, Borst A. Neural Circuit to Integrate Opposing Motions in the Visual Field. Cell. 2015;162(2):351–62. doi: 10.1016/j.cell.2015.06.035 26186189 10.1016/j.cell.2015.06.035

[8] Saleem AB, Longden KD, Schwyn DA, Krapp HG, Schultz SR. Bimodal optomotor response to plaids in blowflies: mechanisms of component selectivity and evidence for pattern selectivity. J Neurosci. 2012;32(5):1634–42. doi: 10.1523/JNEUROSCI.4940-11.2012 22302805 10.1523/JNEUROSCI.4940-11.2012 PMC6703340

[9] Wallach H. Über visuell wahrgenommene Bewegungsrichtung. Psychologische Forschung. 1935;20:325–80.

[10] Carter O, Snyder JS, Fung S, Rubin N. Using ambiguous plaid stimuli to investigate the influence of immediate prior experience on perception. Atten Percept Psychophys. 2014;76(1):133–47. doi: 10.3758/s13414-013-0547-5 24101343 10.3758/s13414-013-0547-5

[11] Hupe JM, Rubin N. The dynamics of bi-stable alternation in ambiguous motion displays: a fresh look at plaids. Vision Res. 2003;43(5):531–48. 12594999 10.1016/s0042-6989(02)00593-x

[12] Borst A, Egelhaaf M, Haag J. Mechanisms of dendritic integration underlying gain control in fly motion-sensitive interneurons. J Comput Neurosci. 1995;2(1):5–18. 8521280 10.1007/BF00962705

[13] Joesch M, Plett J, Borst A, Reiff DF. Response properties of motion-sensitive visual interneurons in the lobula plate of Drosophila melanogaster. Curr Biol. 2008;18(5):368–74. doi: 10.1016/j.cub.2008.02.022 18328703 10.1016/j.cub.2008.02.022

[14] Buchner E. Elementary Movement Detectors in an Insect Visual-System. Biol Cybern. 1976;24(2):85–101. doi: 10.1007/Bf00360648

[15] Qian N, Andersen RA, Adelson EH. Transparent motion perception as detection of unbalanced motion signals. I. Psychophysics. J Neurosci. 1994;14(12):7357–66. 7996181 10.1523/JNEUROSCI.14-12-07357.1994 PMC6576896

[16] Aptekar JW, Shoemaker PA, Frye MA. Figure tracking by flies is supported by parallel visual streams. Curr Biol. 2012;22(6):482–7. doi: 10.1016/j.cub.2012.01.044 22386313 10.1016/j.cub.2012.01.044

[17] Heisenberg M, Wolf R. The sensory-motor link in motion-dependent flight control of flies. Rev Oculomot Res. 1993;5:265–83. 8420552

[18] Götz KG. Optomotor studies of the visual system of several eye mutants of the fruit fly Drosophila. Kybernetik. 1964;2(2):77–92. doi: 10.1007/BF00288561 5833196 10.1007/BF00288561

[19] Heisenberg M, Wolf R. Vision in Drosophila: Genetics of microbehavior. Berlin; New York: Springer-Verlag; 1984. 250 p.

[20] Mayer M, Vogtmann K, Bausenwein B, Wolf R, Heisenberg M. Flight Control during Free Yaw Turns in Drosophila-Melanogaster. J Comp Physiol A. 1988;163(3):389–99. doi: 10.1007/Bf00604014

[21] Theobald JC, Duistermars BJ, Ringach DL, Frye MA. Flies see second-order motion. Curr Biol. 2008;18(11):R464–5. doi: 10.1016/j.cub.2008.03.050 18522814 10.1016/j.cub.2008.03.050

[22] Xi W, Peng Y, Guo J, Ye Y, Zhang K, Yu F, et al Mushroom bodies modulate salience-based selective fixation behavior in Drosophila. Eur J Neurosci. 2008;27(6):1441–51. doi: 10.1111/j.1460-9568.2008.06114.x 18364023 10.1111/j.1460-9568.2008.06114.x

[23] Reichardt W, Wenking H. Optical detection and fixation of objects by fixed flying flies. Naturwissenschaften. 1969;56(8):424–5. doi: 10.1007/BF00593644 10.1007/BF00593644 5362004

[24] Levelt WJ. Note on the distribution of dominance times in binocular rivalry. Br J Psychol. 1967;58(1):143–5. doi: 10.1111/j.2044-8295.1967.tb01068.x 5582864 10.1111/j.2044-8295.1967.tb01068.x

[25] Borsellino A, De Marco A, Allazetta A, Rinesi S, Bartolini B. Reversal time distribution in the perception of visual ambiguous stimuli. Kybernetik. 1972;10(3):139–44. doi: 10.1007/BF00290512 5021011 10.1007/BF00290512

[26] Kovacs I, Papathomas TV, Yang M, Feher A. When the brain changes its mind: interocular grouping during binocular rivalry. Proc Natl Acad Sci U S A. 1996;93(26):15508–11. 8986842 10.1073/pnas.93.26.15508 PMC26435

[27] Murata T, Matsui N, Miyauchi S, Kakita Y, Yanagida T. Discrete stochastic process underlying perceptual rivalry. Neuroreport. 2003;14(10):1347–52. doi: 10.1097/01.wnr.0000077553.91466.41 12876471 10.1097/01.wnr.0000077553.91466.41

[28] Logothetis NK, Leopold DA, Sheinberg DL. What is rivalling during binocular rivalry? Nature. 1996;380(6575):621–4. doi: 10.1038/380621a0 8602261 10.1038/380621a0

[29] Künnapas T. Figural reversal rate and personal tempo. Scand J Psychol. 1969;10(1):27–32. doi: 10.1111/j.1467-9450.1969.tb00004.x 5353395 10.1111/j.1467-9450.1969.tb00004.x

[30] Walker P. Stochastic properties of binocular-rivalry alternations. Percept Psychophys. 1975;18:467–73.

[31] Fox R, Herrmann J. Stochastic properties of binocular rivalry alternations. Percept Psychophys 1967;2:432–46.

[32] Pastukhov A, Garcia-Rodriguez PE, Haenicke J, Guillamon A, Deco G, Braun J. Multi-stable perception balances stability and sensitivity. Front Comput Neurosci. 2013;7:17 doi: 10.3389/fncom.2013.00017 23518509 10.3389/fncom.2013.00017 PMC3602966

[33] Tang S, Juusola M. Intrinsic activity in the fly brain gates visual information during behavioral choices. PLoS ONE. 2010;5(12):e14455 doi: 10.1371/journal.pone.0014455 21209935 10.1371/journal.pone.0014455 PMC3012687

[34] Hausen K. Motion sensitive interneurons in the optomotor system of the fly II. The horizontal cells: Receptive field organization and response characteristics. Biol Cybern. 1982;46(1):67–79. doi: 10.1007/BF00335352

[35] Schacter DL, Gilbert DT, Wegner DM. Psychology. 2 ed: Worth Publishers Inc.; 2011.

[36] Necker L. Observations on some remarkable optical phenomena seen in Switzerland, and on an optical phenomenon which occurs on viewing a figure of a crystal or geometric solid. London Edinburgh Philosophical Magazine and Journal of Science. 1832;1:329–37.

[37] Logothetis NK, Schall JD. Binocular motion rivalry in macaque monkeys: eye dominance and tracking eye movements. Vision Res. 1990;30(10):1409–19. 2247951 10.1016/0042-6989(90)90022-d

[38] Grunewald A, Bradley DC, Andersen RA. Neural correlates of structure-from-motion perception in macaque V1 and MT. J Neurosci. 2002;22(14):6195–207. doi: 20026523 12122078 10.1523/JNEUROSCI.22-14-06195.2002 PMC6757912

[39] Lindsey DT, Todd JT. Opponent motion interactions in the perception of transparent motion. Percept Psychophys. 1998;60(4):558–74. 9628990 10.3758/bf03206046

[40] O'Carroll DC, Barnett PD, Nordstrom K. Local and global responses of insect motion detectors to the spatial structure of natural scenes. J Vis. 2011;11(14):20 doi: 10.1167/11.14.20 22201615 10.1167/11.14.20

[41] Aptekar JW, Keles MF, Lu PM, Zolotova NM, Frye MA. Neurons forming optic glomeruli compute figure-ground discriminations in Drosophila. J Neurosci. 2015;35(19):7587–99. doi: 10.1523/JNEUROSCI.0652-15.2015 25972183 10.1523/JNEUROSCI.0652-15.2015 PMC4429157

[42] Egelhaaf M. On the Neuronal Basis of Figure-Ground Discrimination by Relative Motion in the Visual System of the Fly I. Behavioural Constraints Imposed on the Neuronal Network and the Role of the Optomotor System. Biol Cybern. 1985;52:123–40.

[43] Fox JL, Aptekar JW, Zolotova NM, Shoemaker PA, Frye MA. Figure-ground discrimination behavior in Drosophila. I. Spatial organization of wing-steering responses. J Exp Biol. 2014;217(Pt 4):558–69. doi: 10.1242/jeb.097220 24198267 10.1242/jeb.097220 PMC3922833

[44] Bahl A, Ammer G, Schilling T, Borst A. Object tracking in motion-blind flies. Nat Neurosci. 2013;16(6):730–8. doi: 10.1038/nn.3386 23624513 10.1038/nn.3386

[45] Fenk LM, Poehlmann A, Straw AD. Asymmetric processing of visual motion for simultaneous object and background responses. Curr Biol. 2014;24(24):2913–9. doi: 10.1016/j.cub.2014.10.042 25454785 10.1016/j.cub.2014.10.042

[46] Theobald JC, Shoemaker PA, Ringach DL, Frye MA. Theta motion processing in fruit flies. Front Behav Neurosci. 2010;4 doi: 10.3389/fnbeh.2010.00035 20700393 10.3389/fnbeh.2010.00035 PMC2918350

[47] Silies M, Gohl DM, Clandinin TR. Motion-detecting circuits in flies: coming into view. Annu Rev Neurosci. 2014;37:307–27. doi: 10.1146/annurev-neuro-071013-013931 25032498 10.1146/annurev-neuro-071013-013931

[48] Koenig S, Wolf R, Heisenberg M. Vision in Flies: Measuring the Attention Span. PLoS ONE. 2016;11(2):e0148208 doi: 10.1371/journal.pone.0148208 26848852 10.1371/journal.pone.0148208 PMC4744059

[49] Koenig S. Spatially selective visual attention in Drosophila melanogaster [Dissertation]. University of Wuerzburg: University of Wuerzburg; 2016.

[50] Wolf R, Heisenberg M. On the Fine-Structure of Yaw Torque in Visual Flight Orientation of Drosophila-Melanogaster .2. A Temporally and Spatially Variable Weighting Function for the Visual-Field (Visual-Attention). J Comp Physiol. 1980;140(1):69–80. doi: 10.1007/BF00613749

[51] Mueller TJ, Blake R. A Fresh Look at the Temporal Dynamics of Binocular-Rivalry. Biol Cybern. 1989;61(3):223–32. doi: 10.1007/Bf00198769 2765591 10.1007/BF00198769

[52] Long GM, Toppino TC. Enduring interest in perceptual ambiguity: alternating views of reversible figures. Psychol Bull. 2004;130(5):748–68. doi: 10.1037/0033-2909.130.5.748 15367079 10.1037/0033-2909.130.5.748

[53] Wilson HR. Computational evidence for a rivalry hierarchy in vision. Proc Natl Acad Sci U S A. 2003;100(24):14499–503. doi: 10.1073/pnas.2333622100 14612564 10.1073/pnas.2333622100 PMC283620

[54] Maesani A, Ramdya P, Cruchet S, Gustafson K, Benton R, Floreano D. Fluctuation-Driven Neural Dynamics Reproduce Drosophila Locomotor Patterns. PLoS Comput Biol. 2015;11(11):e1004577 doi: 10.1371/journal.pcbi.1004577 26600381 10.1371/journal.pcbi.1004577 PMC4657918

[55] Maye A, Hsieh CH, Sugihara G, Brembs B. Order in spontaneous behavior. PLoS ONE. 2007;2(5):e443 doi: 10.1371/journal.pone.0000443 17505542 10.1371/journal.pone.0000443 PMC1865389

[56] Guo A, Li L, Xia SZ, Feng CH, Wolf R, Heisenberg M. Conditioned visual flight orientation in Drosophila: dependence on age, practice, and diet. Learn Mem. 1996;3(1):49–59. doi: 10.1101/lm.3.1.49 10456076 10.1101/lm.3.1.49

